# Effect of pH and medium composition on chain elongation with *Megasphaera hexanoica* producing C_4_-C_8_ fatty acids

**DOI:** 10.3389/fmicb.2023.1281103

**Published:** 2023-11-02

**Authors:** Carla Fernández-Blanco, María C. Veiga, Christian Kennes

**Affiliations:** Chemical Engineering Laboratory, Faculty of Sciences and Interdisciplinary Centre of Chemistry and Biology – Centro Interdisciplinar de Química y Biología (CICA), BIOENGIN Group, University of A Coruña, Coruña, Spain

**Keywords:** *Megasphaera hexanoica*, lactate-based chain elongation, *n*-caproate, *n*-caprylate, *iso*-valerate

## Abstract

**Introduction:**

Chain elongation technology, which involves fermentation with anaerobic bacteria, has gained attention for converting short and medium chain substrates into valuable and longer-chain products like medium chain fatty acids (MCFAs). In the recent past, the focus of studies with pure chain elongating cultures was on species of other genera, mainly *Clostridium kluyveri*. Recently, other chain elongators have been isolated that deserve further research, such as *Megasphaera hexanoica*.

**Methods:**

In this study, batch studies were performed in bottles with two different media to establish the optimal conditions for growth of *M. hexanoica*: (a) a medium rich in different sources of nitrogen and (b) a medium whose only source of nitrogen is yeast extract. Also, batch bioreactor studies at pH values of 5.8, 6.5 and 7.2 were set up to study the fermentation of lactate (i.e., electron donor) and acetate (i.e., electron acceptor) by *M. hexanoica*.

**Results and discussion:**

Batch bottle studies revealed the yeast extract (YE) containing medium as the most promising in terms of production/cost ratio, producing *n*-caproate rapidly up to 2.62 ± 0.24 g/L. Subsequent bioreactor experiments at pH 5.8, 6.5, and 7.2 confirmed consistent production profiles, yielding C_4_-C_8_ fatty acids. A fourth bioreactor experiment at pH 6.5 and doubling both lactate and acetate concentrations enhanced MCFA production, resulting in 3.7 g/L *n*-caproate and 1.5 g/L *n*-caprylate. H_2_ and CO_2_ production was observed in all fermentations, being especially high under the increased substrate conditions. Overall, this study provides insights into *M. hexanoica*’s behavior in lactate-based chain elongation and highlights optimization potential for improved productivity.

## 1. Introduction

The production of high value-added products such as medium MCFAs through the chain elongation cycle, based on the reverse β-oxidation pathway, has been recently reported. This bioprocess is usually carried out with mixed cultures (Kucek et al., 2016a,b; Reddy et al., 2018; Spirito et al., 2018), since they can take advantage of a wide range of substrates and nutrients, there result in synergic interactions among the species, and the process does not require working under sterile conditions. Even so, in recent times, the available literature on the production of MCFAs with pure cultures has been limited compared to mixed cultures (Jeon et al., 2010; Yin et al., 2017; Candry et al., 2018; San-Valero et al., 2020). The best known chain elongating microorganism is *Clostridium kluyveri*, which has been shown to produce *n*-butyrate, *n*-caproate and occasionally *n*-caprylate, both in batch and continuous mode from ethanol and acetate (Gildemyn et al., 2017; San-Valero et al., 2019). Besides, the production of odd-chain fatty acids such as *n*-valerate and *n*-heptanoate using ethanol and propionate have been reported as well (Candry et al., 2020).

In recent developments, pure strains belonging to the *Clostridia* class have been isolated and demonstrated to perform lactate fermentation, predominantly yielding *n*-caproate and *iso*-butyrate via the chain elongation process (Liu et al., 2020). On the other hand, species of the genus *Megasphaera* have also been shown to be able to produce high concentrations of *n*-caproate and other MCFAs through the chain elongation cycle (Andersen et al., 2015; Angenent et al., 2016; Kucek et al., 2016c; Sträuber et al., 2018). For instance, *Megasphaera elsdenii* was isolated from sheep rumen contents (Elsden and Lewis, 1953) and its main fermentation end products are acetate, propionate, *n*-butyrate, *n*-valerate, *n*-caproate, H_2_ and CO_2_ in different proportions depending on the substrate, which can be DL-lactate and sugars such as glucose, fructose and sucrose (Elsden et al., 1956). The good performance of *M. elsdenii* in producing *n*-caproate through the chain elongation cycle has been documented in several studies in the last decade (Choi et al., 2013; Weimer and Moen, 2013; Nelson et al., 2017). Moreover, new strains have been recently isolated such as *Megasphaera hexanoica*, another strain belonging to this genus that deserves special attention for its promising ability to produce bioproducts via chain elongation. It is a strictly anaerobic, gram-negative, non-spore-forming microorganism isolated from cattle rumen which thrives at temperatures between 30 to 40°C and pH levels ranging from 5.5 to 7.5 (Jeon et al., 2017). Its main fermentation end products are acetate, *n*-butyrate, *iso*-butyrate, *n*-valerate, *iso*-valerate, *n*-caproate, *n*-heptanoate, and *n*-caprylate, as well as H_2_ and CO_2_ and it is able to metabolize mainly fructose, and to a weaker or variable extent, galactose, mannose, D, L-lactate and gluconate. Kang et al. (2022) proved that this bacterium has a good performance producing MCFAs using lactate as electron donor and evaluated the effect of using acetate and/or *n*-butyrate as electron acceptor, yielding 8.9 g/L *n*-caproate from 10 g/L lactate, 11 g/L *n*-butyrate and 6 g/L acetate, approximately. When comparing the use of fructose and lactate as electron donors, it was found that lactate resulted in a specific titer and specific productivity that were more than three times higher than fructose. Moreover, the simultaneous utilization of fructose and lactate, both as electron donors, led to a maximum *n*-caproate concentration of 13.8 g/L. For this to happen, the substrate concentrations used were 6 g/L acetate, and 10 g/L of fructose, lactate, and *n*-butyrate, with an extra pulse of lactate up to almost 6 g/L prior to its complete depletion. On the other hand, it has been shown that propionate as a substrate in this strain is involved in the formation of odd-chain fatty acids such as *n*-valerate and *n*-heptanoate (Kim et al., 2019). In a medium composed of 20 g/L fructose, 2.42 g/L acetate and 18.91 g/L propionate, concentrations of up to 9.48 g/L *n*-valerate, 2.48 g/L *n*-heptanoate and 0.12 g/L *n*-caproate were achieved, thus demonstrating that the selectivity of the process can be steered towards certain products (odd chains) thanks to the selection of the substrate in the chain elongation. Likewise, different combinations of carboxylates as electron acceptors were studied using fructose as electron donor (Jeon et al., 2016). 5.7 g/L *n*-valerate, 1.5 g/L *n*-caproate, 2.7 g/L *n*-heptanoate and 0.2 g/L *n*-caprylate were obtained from fructose (20 g/L), acetate (6 g/L) and propionate (7.4 g/L), whereas 0.3 g/L *n*-valerate, 9.7 g/L *n*-caproate and 0.6 g/L *n*-caprylate were detected from fructose (20 g/L), acetate (6 g/L) and *n*-butyrate (11.6 g/L). Finally, this strain was also investigated in a co-culture with *n*-butyrate-producing *Clostridium* strains, which would be elongated into *n*-caproate by *M. hexanoica* (Kim et al., 2018). For this purpose, the two types of strains were cultured in two submerged hollow-fiber membrane bioreactors resulting in *n*-caproate concentrations of up to 10.08 g/L, with a productivity of 0.69 g/(L·h) and a *n*-caproate yield of 0.55 g/g sugar.

However, since only one study is available to date that analyzes chain elongation with this microorganism from lactate (Kang et al., 2022), it is a largely unexplored horizon. The use of lactate is interesting as an electron donor in chain elongation for several reasons. Firstly, its production cost from waste biomass by anaerobic fermentation is relatively low (Wu et al., 2019); secondly, during some food waste degradation, lactate production has been reported to account for 74.4 to 96.8% of the total acid content (Gu et al., 2018). On the other hand, acetate has been used extensively as an electron acceptor in chain elongation throughout the literature and in addition, its market price is much lower than other carboxylates, such as *n*-butyrate and longer chain acids (Zhao et al., 2022). For this same reason, it would be worthwhile to investigate whether the addition of different nitrogen sources and the fermentation pH could have any impact on the strain’s performance using lactate and acetate. It was hypothesized that if the bacterium is able to maintain high activity over a wide pH range, it would have some advantage over other pH-sensitive chain elongators such as *C. kluyveri*. Furthermore, *M. hexanoica* exhibits a wider array of potential substrates beyond lactate, including fructose, and to a lesser extent, galactose, and gluconate (Jeon et al., 2017). Conversely, *C. kluyveri* does not ferment sugars and primarily relies on ethanol as its substrate (i.e., electron donor). Thus, this work aimed to examine the growth and production of MCFAs from a pure culture of *M. hexanoica* using only lactate and acetate as electron donor and acceptor and thereby contributing to the state of the art. For this purpose, the behavior of the strain was assessed in a bottle study using two media with different degrees of enrichment and afterwards, the growth at pH values of 5.8, 6.5 and 7.2 in bioreactors was evaluated. Likewise, after verifying the effect of pH, an attempt was made to optimize *n*-caproate production by changing the fermentation strategy.

## 2. Materials and methods

### 2.1. Microorganism and culture media

*M. hexanoica* DSM 106893 was acquired as a freeze-dried pellet from the Deutsche Sammlung von Mikroorganismen und Zellkulturen GmbH (Braunschweig, Germany). Initially, the strain was activated in an enriched medium containing fructose and later, after successive inoculations, it was adapted to a medium with lactate and acetate, and the composition described below. All incubations were done at 37°C and under constant shaking conditions at 100 rpm.

The enriched medium used to grow *M. hexanoica* contained (per liter distilled water): yeast extract, 10 g; tryptone, 5 g; meat peptone, 5 g; meat extract, 5 g; K_2_HPO_4_, 2 g; cysteine-HCl, 0.5 g; saline solution, 40 mL; Na-Acetate·3H_2_O, 5.04 g; L-(+)-lactic acid 90%, 9.25 mL.

In the only YE-medium, tryptone, meat peptone and meat extract were omitted. In the medium with no enrichment, yeast extract was also omitted along with the other components just mentioned before.

The composition of the saline solution (per liter distilled water) was as follows: CaCl_2_·2H_2_O, 0.25 g; MgSO_4_·7H_2_O, 0.5 g; K_2_HPO_4_, 1 g; KH_2_PO_4_, 1 g; NaHCO_3_, 10 g; NaCl, 2 g.

The pH of the medium was always adjusted to 6.5, unless otherwise indicated.

### 2.2. Batch bottles assay

To establish the optimum growth medium for *M. hexanoica* and the effect of the addition of certain nutrients, two media with different degrees of enrichment were tested. To do so, after dissolving all the components in 40 mL of distilled water, the serum bottles were purged with pure nitrogen both in the liquid and in the headspace for at least 6 min. Simultaneously, the pH of the solution was adjusted to 6.5, using either 2 M HCl or 2 M NaOH. Then, the bottles were sealed with butyl rubber septa and aluminum crimps and autoclaved for 20 min at 121°C. This trial was carried out in triplicate.

Each bottle was inoculated with 10% (v/v) seed culture of *M. hexanoica* in the early exponential growth phase as follows: from a seed inoculum containing the enriched medium, two consecutive transfers were made to a bottle with YE-only medium. Once the bacteria entered the early exponential growth phase, a third inoculation was made to each of the bottles under study. This way, the additional carbon and nitrogen sources in the enriched medium were diluted 1,000-fold and the same seed inoculum was also used for all bottles.

All bottles were sampled daily, at least once. Thus, 1 mL of the liquid phase was withdrawn for pH measurement, OD_600 nm_ determination and HPLC analysis and 2 mL of gaseous sample (headspace) for H_2_ and CO_2_ analysis by gas chromatography.

For the calculation of the growth rate, at least three OD_600nm_ data points of the exponential growth phase were taken and plotted on an exponential scale versus time. The growth rate (μ) was estimated in h^−1^ as shown in Eq. 1.


(1) $$y={Ae}^{\mu \left(h^{-1}\right)x}$$

### 2.3. Bioreactor studies

The experiments were conducted using 2 L Eppendorf BIOFLO 120 bioreactors, with a working volume of 1.2 L, and at a constant temperature of 37°C (Eppendorf AG, Hamburg, Germany). For all experiments, the previously described medium containing only yeast extract was introduced into the bioreactor, excluding D-(+)-lactic acid, and then autoclaved at 121°C for 20 min. After cooling down to room temperature (20°C), the reactor medium was subjected to 1.5-h flushing with pure nitrogen using a sparger, while stirring at 100 rpm, to create anaerobic conditions. Subsequently, D-(+)-lactic acid was added aseptically and anaerobically to the reactor through a 0.22 μm PTFE sterile filter. Based on the theoretical pH range of growth for *M. hexanoica* (5.5–7.5), it was decided to study the behavior of the strain under conditions of pH 5.8, 6.5, and 7.2 (i.e., with 0.7 pH unit interval between the tested pH values) to determine whether *M. hexanoica* maintains a constant activity over a wide pH range. The pH adjustment was done by adding HCl 1 M or NaOH 1 M via peristaltic pumps. The control unit used was programmed so that the pH setpoint would only admit an error of 0.02 units, so that when this limit was exceeded both above and below, the peristaltic pumps were activated, and pH was corrected. A seed inoculum of *M. hexanoica*, in early exponential growth phase (10% v/v, 120 mL), was introduced into the reactor. The pH was continuously monitored and adjusted throughout the experiment. To prevent overpressure in the system, a gas outlet port was used, from which gaseous samples were also taken. Whenever excessive foaming occurred during the purging process, a few drops of antifoam were dispensed into the synthetic medium, which made the foam disappear in a matter of seconds. In addition, a MilliGascounter (Ritter) was placed at the outlet of each reactor to measure the amount of H_2_ and CO_2_ flow at the outlet.

One or two liquid samples were aseptically collected from the bioreactors on a daily basis. These samples were then used for optical density measurements at a wavelength of 600 nm (OD_600nm_) and for analysis of metabolites. Also, gas samples of 1 mL were taken from the outlet of the bioreactors, in duplicate, to determine the levels of H_2_ and CO_2_.

The *n*-caproate selectivity for all conditions was calculated in % (mol/L) as follows (Eq. 2).


(2) $$nC_{6}\;\mathrm{selectivity}\;\left(\%\right)=\frac{{\left[nC_{6}\right]}_{\mathrm{end}}\;\left(\frac{\mathrm{mol}}{L}\right)}{\left(\begin{array}{l} {\left[nC_{4}\right]}_{\mathrm{end}}+{\left[nC_{5}\right]}_{\mathrm{end}}+{\left[iC_{5}\right]}_{\mathrm{end}}\\ +\;{\left[nC_{6}\right]}_{\mathrm{end}}+{\left[iC_{6}\right]}_{\mathrm{end}}+{\left[nC_{8}\right]}_{\mathrm{end}} \end{array}\right)\left(\frac{\mathrm{mol}}{L}\right)}\times 100$$

### 2.4. Analytical methods

Bacterial growth was estimated by measuring the optical density at a wavelength of 600 nm (OD_600nm_) of the non-filtered samples with a cuvette (pathlength of 10 mm) using a UV–visible spectrophotometer (Hitachi, Model U-200, Pacisa & Giralt, Madrid, Spain). These measurements, obtained in absorbance units, serve as indicators of the bacteria’s growth phase, with higher values corresponding to increased bacterial activity (and consequently higher MCFAs productivity).

The concentrations of carboxylates (C_2_-C_6_) were determined using a High Performance Liquid Chromatography (HPLC) with an HP1100 system (Agilent Co., Palo Alto, United States). The HPLC system was equipped with an Agilent Hi-Plex Column (300 × 7.7 mm) and both a diode array detector (DAD) and a refractive index detector (RID) maintained at 50°C. A 0.005 M H_2_SO_4_ solution was utilized as mobile phase, with a flow rate of 0.80 mL/min and a column temperature of 45°*C. prior* to analysis, all liquid samples were filtered through 0.22 μm PTFE syringe filters.

Heptanoate (C_7_) and caprylate (C_8_) concentrations were detected on a gas chromatograph equipped with a flame ionization detector (GC-FID), with the internal standard method, using 4-methylpentanoic acid as the internal standard. Prior to analysis, the aqueous samples from the reactor were subjected to an extraction process with dichloromethane in 10 mL tubes, which was the solvent used for gas chromatography in this case. For this, 2 mL sample, 2 mL dichloromethane, 0.4 g NaCl, 0.4 mL of the internal standard solution, and 0.5 mL H_2_SO_4_ 50% were introduced in the tube. They were then shaken at 2000 rpm for 10 min and centrifuged at 3000 rpm for 3 min. Finally, the organic phase was withdrawn and placed in the corresponding vial. The equipment used was a Thermo Scientific TRACE 1300 GC equipped with a DB-FATWAX Ultra Inert column with a length of 30 m, diameter 0.250 mm and film thickness 0.25 μm was used. The temperature of both the detector and the injector was maintained constant at 270°C and 280°C, respectively, whereas the program used in the oven was as follows: first, the temperature was held at 120°C for 1 min; then it was increased, with a ramp of 5°C/min, up to 140°C and held for 3 min; then, the temperature was increased again, with a ramp of 15°C/min, up to 230°C and held for 1 min. Air, makeup gas (nitrogen) and hydrogen flow rates were, respectively, 350 mL/min, 40 mL/min and 35 mL/min. The carrier gas was helium at a flow rate of 62 mL/min with a split ratio of 50.

H_2_ and CO_2_ concentrations were determined by gas chromatography (GC, Agilent Technologies, Madrid, Spain). The GC used for H_2_ measurements was composed of a thermal conductivity detector (TCD) and a 15-m HP-PLOT Molecular Sieve 5 A column (0.53 mm ID, 50 μm film thickness). The equipment used for CO_2_ determinations was a HP 5890 gas chromatograph (GC, Agilent Technologies, Madrid, Spain), containing a Porapak Q 80/100 (inox) column (2 m × 1/8′) connected to a thermal conductivity detector (TCD). The injection, oven and detection temperatures were kept at 90, 25 and 100°C, respectively. In both cases, helium was used as carrier gas.

## 3. Results and discussion

### 3.1. Medium composition study

This study aimed at evaluating the effect of the presence or absence of certain nutrients in the culture medium on the growth and production of MCFAs by *M. hexanoica*. Therefore, the strain was inoculated in two media with different substrates as carbon source: (a) enriched medium: with yeast extract, tryptone, meat peptone, meat extract, lactate, and acetate; and (b) only YE-medium: with yeast extract, lactate, and acetate.

There is only one work in the literature reporting the ability of *M. hexanoica* to ferment lactate and acetate to produce mainly *n*-caproate (Kang et al., 2022). In this study, the strain was grown in presence of 10 g/L YE as the sole source of nitrogen, but there are a few studies in which this strain has been grown on a medium richer in nitrogen sources (Jeon et al., 2017; Kim et al., 2018, 2019; Park et al., 2020). However, whether this additional nitrogen source could have a positive impact on the growth of *M. hexanoica* during chain elongation from lactate and acetate has not ever been reported. It is known that yeast extract, meat extract, meat peptone, tryptone, are organic sources of nitrogen necessary for bacterial growth, since this element is involved in protein synthesis processes, nucleic acids, and energy generation, among others. Authors such as Yang et al. (2018) have also stated that yeast extract also provides essential nutrients, which in the first instance should stimulate further growth and activity of the strain. Therefore, in order to perform the reactor studies detailed in later sections, it was desired to determine if indeed, the addition of extra nitrogen sources would improve the productivity or kinetics of the fermentation during chain elongation with lactate and acetate. San-Valero et al. (2020) observed that in a medium without yeast extract the optical densities achieved with *C. kluyveri* were significantly lower, as well as the amount of *n*-caproate produced, than in other experiments in the presence of YE. For this same reason, a medium without the minimum nitrogen requirements for the cells would be undesirable. The authors hypothesized that, if YE is present in the medium, the strain uses it for growth, while the electron donor and acceptor are used for the chain elongation process.

**Figure 1:**
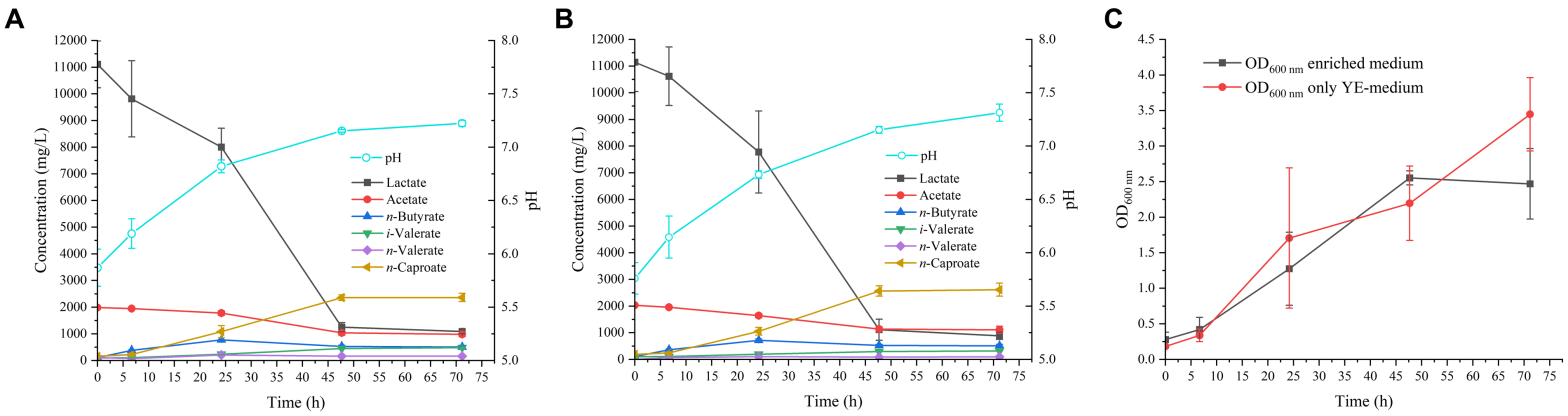
Profile of MCFAs production by *M. hexanoica* in **(A)** enriched medium; **(B)** only YE-medium. **(C)** Represents the growth curve of the strain (in OD 600 nm values) as a function of time for the two tested media.

One of the experiments undertaken by Kang et al. (2022) in batch mode produced 8.9 g/L *n*-caproate from approximately 10 g/L lactate (0.11 mol), 11 g/L *n*-butyrate (0.12 mol) and 6 g/L acetate (0.10 mol), which corresponds to an electron donor:acceptor ratio of approximately 0.5. On the other hand, other authors have reported that an excess of lactate over acetate is necessary to efficiently accomplish the chain elongation process towards the production of MCFAs with other pure cultures, such as *Caproicibacterium lactatifermentans* (Wang et al., 2022). For instance, Tang et al. (2022) observed that during lactate-based chain elongation with a mixed culture the highest concentrations of *n*-caproate (11.02 mmol/L, 1.28 g/L) occurred at a lactate:acetate molar ratio of 3:1 and that ratios below this led to an accumulation of shorter chain carboxylates (i.e., *n*-butyrate). This phenomenon was also previously described in ethanol-based chain elongation with *C. kluyveri* (Fernández-Blanco et al., 2022). Also, it is indeed factual that results presented in Kang et al. (2022) indicate that the utilization of *n*-butyrate promotes the production of *n*-caproate over acetate. Nevertheless, acetate is put forward in this context as an electron acceptor for several compelling reasons: (a) due to economic considerations, as stated before; (b) stemming from the desire to yield the most elongated products feasible from the shortest carbon source (i.e., C_2_); and (c) given that acetate can be produced from acetogenic bacteria utilizing C_1_ gases, which offers the potential for a synergistic coupling of syngas fermentation and chain elongation with *M. hexanoica*. Conversely, the production of *n*-butyrate is confined to a very limited number of acetogenic bacteria, such as *C. carboxidivorans* (Fernández-Naveira et al., 2019). These are the reasons, besides the fact that there are hardly any data available in the literature on lactate fermentation with *M. hexanoica*, i.e., it is a process that requires optimization, for which all the experiments presented in this work were run with a fixed initial lactate:acetate molar ratio of 3:1. The production profile of MCFAs as well as the variation of the pH of the culture medium can be seen in Figures 1A,B.

After inoculation of *M. hexanoica*, it was observed that the strain managed to grow with almost no lag phase in the two different media. Lactate and acetate consumptions and the subsequent production of MCFAs were stopped in both media after 71 h. Table 1 shows a comparison of the results of both fermentations, with the enriched medium and the YE-only medium. There were no significant differences regarding lactate and acetate use, yet the maximum OD_600nm_ (Figure 1C) and the maximum *n*-caproate concentration reached were slightly higher in the YE-only medium. The second most abundant metabolite obtained was *n*-butyrate, reaching final concentrations of 0.51 ± 0.032 g/L, and 0.51 ± 0.051 g/L, respectively, followed by *iso*-valerate, with 0.48 ± 0.019 g/L, and 0.32 ± 0.003 g/L, respectively. Unlike ethanol-based chain elongation, when lactate is used as a substrate, CO_2_ is released in addition to H_2_. This is due to the oxidation of lactate to pyruvate (3 carbons), which is then further oxidized to acetyl-CoA (2 carbons) by pyruvate decarboxylase, with the consequent generation of CO_2_ (Cavalcante et al., 2017; Wu et al., 2018). In these experiments, H_2_ and CO_2_ production was also observed in the headspace of the bottles, being similar in all cases: 13.2 ± 3.8% and 17.5 ± 3.2% (% v/v, final H_2_ and CO_2_, respectively, in the enriched medium), 10.5 ± 0.94% and 18.4 ± 1.7% (% v/v, final H_2_ and CO_2_, respectively, in the only YE-medium).

**Table 1:** Comparison of results after culturing *M. hexanoica* in bottles using two media of different composition.

	Max. OD_600 nm_	*μ* (h^−1^)	Lactate consumption (%)	Acetate consumption (%)	Max. *n*-caproate (g/L)
Enriched medium	2.55 ± 0.10 (48 h)	0.0446 ± 0.00731	90.4 ± 0.36	50.6 ± 3.8	2.32 ± 0.16
YE-only medium	3.45 ± 0.52 (71 h)	0.0460 ± 0.0128	92.1 ± 0.54	45.4 ± 6.1	2.62 ± 0.24

On the other hand, the initial pH in both conditions was 5.8 (adjusted to 6.5 before autoclaving and lowered as a result of acidification of the acids present in the inoculum), while the final pH remained around 7.3. This pattern of gradual increase in pH is normal during lactate fermentation, given the lower lactic acid pK_a_ of 3.86, compared to the pK_a_ value of the MCFAs produced, 4.76 (Lonkar et al., 2016; Lübeck and Lübeck, 2019). This is indicative of lactate being a stronger acid than MCFAs since the outcome of the lactate gradual disappearance from the medium as it is transformed into products, is an increment in pH. Such a successive increase in pH also improves the solubility of carboxylic acids present in the broth, as they are mostly in their dissociated form (i.e., carboxylates), and therefore, the risk of toxicity due to the accumulation of non-dissociated acids is minimized.

In conclusion, given the resemblance of the data in terms of kinetics and production of both soluble (MCFAs) and gaseous (H_2_, CO_2_) compounds, and in view of the fact that the addition of meat extract, meat peptone and tryptone do not improve any of these aspects compared to a medium with only YE as nitrogen source, it was decided to choose the YE-only medium to grow *M. hexanoica* in the subsequent bioreactor experiments. It is advisable to use a medium with the lowest possible degree of enrichment, in order to reduce costs in the event of scaling up the process (Grootscholten et al., 2013).

### 3.2. Study of pH effect in bioreactors

#### 3.2.1. Intermediate pH of 6.5

Studies available in the literature indicate that *M. hexanoica* has a growth pH ranging between 5.5 and 7.5 (Jeon et al., 2017). However, there is no report available on the effect of pH on the production of MCFAs with this microorganism. Therefore, the purpose of this study was to assess such effect. For this, a pH value of 6.5 established as optimum (Jeon et al., 2017), a slightly more acidic pH (5.8) and a slightly more basic pH (7.2) were also tested.

**Figure 2:**
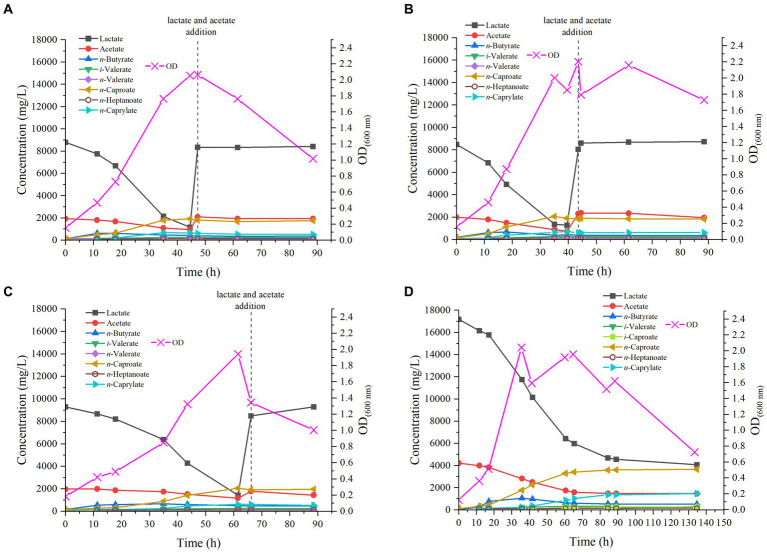
Profile of MCFAs production by *M. hexanoica* from lactate and acetate in the four bioreactor experiments tested: **(A)** pH 6.5; **(B)** pH 5.8; **(C)** pH 7.2; **(D)** pH 6.5 with double substrate of lactate and acetate.

In the reactor started at pH 6.5, virtually no lag phase was observed after inoculation of *M. hexanoica*, with the OD_600nm_ increasing from 0.154 to 0.470 over the first 11 h of experiment, indicative that the inoculum was very active in the exponential growth phase. Chain elongation activity was evident and so was lactate consumption, dropping very rapidly from 8.8 g/L to 7.8 g/L over the same time period. However, acetate consumption was much slower throughout the experiment, with 1.1 g/L left over, in fact, from the initial 1.9 g/L, prior to the addition of the substrate supplement (Figure 2A). The much faster consumption of lactate compared to acetate may be due to an excess of acetate in the medium that could perhaps be solved in the future by optimizing lactate:acetate ratios for this microorganism.

*n*-Butyrate and *n*-caproate production also increased during the first few hours after inoculation, with concentrations of 0.62 g/L and 0.50 g/L, respectively (*t* = 11 h). From this point on, *n*-butyrate production reached a plateau, and its concentration started to decrease, a sign that part of it was being used for *n*-caproate accumulation. This is a typical behavior in chain elongation in which there is an excess of electron donor over acceptor (Kucek et al., 2016b). At the same time, the maximum concentration of *n*-caprylate was detected 35 h after start-up, with a value of 0.69 g/L. In the end, the maximum *n*-caproate concentration was 1.9 g/L. Interestingly, odd chains were also found, such as *n*-heptanoate, whose maximum concentration was 0.14 g/L, *iso*-valerate (0.26 g/L) and *n*-valerate (0.12 g/L). Trace concentrations of *iso*-caproate were also found (data not shown). On the other hand, there is an acrylate pathway that directly competes with the chain elongation that steers carbon flow from lactate to propionate and thus decreases the selectivity of MCFAs (Wu et al., 2018). This pathway may be effectively suppressed with low pH and low substrate concentrations (Xie et al., 2021). Nevertheless, *M. hexanoica* cannot utilize the acrylate pathway as opposed to *M. elsdenii* (Prabhu et al., 2012), based on whole genome sequence data (Kang et al., 2022), and although propionate concentrations were detected occasionally during the experiments, they either remained constant or never exceeded 300 mg/L.

At 47 h, just before lactate was completely depleted (with 1.2 g/L leftover), the initial concentrations of lactate and acetate were adjusted again, adding them under sterile and anaerobic conditions, so as to avoid decay of bacterial activity and to further stimulate the production of MCFAs. Nonetheless, after doing this the OD_600nm_ started to drop, which is a sign of the cellular activity decay, and the lactate and acetate consumption stopped as well as productivity. This behavior has been previously reported in the literature, in chain elongation studies with *C. kluyveri* (San-Valero et al., 2019) and may be due to the entry of air into the reactor or to the stress on the cells due to the abrupt variation in pH when lactate was added, acidifying the medium well below the optimum pH, even if this was only momentarily. Another hypothesis is that the nutrients in the medium (i.e., mineral salts, organic nitrogen sources, etc.) have already been fully utilized and therefore the addition of lactate and acetate alone would not be sufficient to stimulate further cellular activity.

#### 3.2.2. Slightly acidic pH of 5.8

When tested at pH 5.8, the pattern followed by the strain was quite similar to that observed at pH 6.5, since *M. hexanoica* hardly showed any lag phase, gradually increasing the OD_600nm_ up to a maximum of 2.0, after 35 h (Figure 2B). Lactate consumption, as in the previous case, was immediate, evidenced by the drop in lactate concentration from 8.5 g/L to 6.8 g/L over the first 11 h of experimental run. Finally, the maximum concentrations produced of *n*-butyrate, *iso*-valerate, *n*-valerate, *n*-caproate, *n*-heptanoate and *n*-caprylate were, respectively, 0.68 g/L, 0.30 g/L, 0.12 g/L, 2.1 g/L, 0.14 g/L and 0.70 g/L. Also, *iso*-caproate concentrations, below 50 mg/L, were detected during fermentation. Once again, no MCFA production was noted after refreshing the substrate concentrations at *t* = 43.5 h, before lactate depletion.

#### 3.2.3. Slightly alkaline pH of 7.2

In case of the reactor working at the most alkaline pH of those tested, *M. hexanoica* also managed to grow immediately after inoculation, although in a slightly slower manner than in the two previous cases, thus reaching its maximum OD_600nm_ of 2.0 at *t* = 61.5 h (Figure 2C). Lactate consumption was progressive from the first day, and when it reached 1.4 g/L, as in the previous cases, lactate and acetate concentrations were readjusted unsuccessfully, with the aim of continuing fermentation (*t* = 66 h). In this case, the highest concentrations of *n*-butyrate, *iso*-valerate, *n*-valerate, *n*-caproate, *n*-heptanoate, and *n*-caprylate were, respectively, 0.66 g/L, 0.26 g/L, 0.10 g/L, 2.1 g/L, 0.13 g/L, and 0.62 g/L. Likewise, trace concentrations of *iso*-caproate were measured in the fermentation broth.

If the results obtained at three different pH values are evaluated jointly and compared, it can be stated that *M. hexanoica* maintains a high level of activity in a pH range of at least 1.4 units. This offers great versatility and opens up a wide range of possibilities for co-culturing *M. hexanoica* in a microbiome, performing chain elongation in mildly acidic conditions (pH ~ 5.5) in order to inhibit acetoclastic methanogens (Spirito et al., 2018). In contrast, other microorganisms such as *C. kluyveri* have shown serious constraints in staying active at pH around 5 (Ganigué et al., 2016), and its pH activity range is not really wide, making it challenging to find a suitable working pH in co-cultures (Fernández-Blanco et al., 2022; Parera Olm and Sousa, 2023). So far, only one study of co-culturing *M. hexanoica* with other strains is available in the literature. Kim et al. (2018) developed a system with submerged hollow-fiber membrane bioreactors in which *C. tyrobutyricum* BAS7 and *M. hexanoica* were grown in separate chambers. *C. tyrobutyricum* BAS7 produced 15.8 g/L butyrate from sucrose, and this *n*-butyrate-containing medium was filtered and propelled into the chamber containing *M. hexanoica* to produce up to 10.08 g/L *n*-caproate, with a productivity of 0.69 g/(L·h).

In general, it can be concluded that the production profile of MCFAs, especially the trends observed in the formation of *n*-caproate, are quite similar at the studied pHs of 5.8, 6.5 and 7.2. Also, the strategy of reestablishing substrate concentrations just before depletion was not satisfactory in any of the three reactors as indicated above. The main dissimilarities are due to slight kinetic differences and small fluctuations of OD_600nm_, the maximum OD_600nm_ value being nevertheless of the same order in all cases. Besides, in the three pH conditions studied, the production of odd-chain MCFAs from lactate and acetate, such as *n*-valerate, *iso*-valerate (C_5_), and *n*-heptanoate (C_7_), could be observed. In other investigations with the same strain, high concentrations of *n*-valerate (9.48 g/L) and *n*-heptanoate (2.48 g/L) have also been detected, whereas the substrates used were fructose, acetate and propionate (Kim et al., 2019). In another recent study, the production of MCFAs by *M. hexanoica* from lactate as electron donor and acetate and/or *n*-butyrate as electron acceptor was analyzed for the first time (Kang et al., 2022). The authors have only qualitatively detected odd chains such as *n*-valerate and *n*-heptanoate so that it seems that this is the first time that the production of odd-chain MCFAs from lactate and acetate by *M. hexanoica* is quantified so far.

In short, *M. hexanoica* has the potential to catalyze lactate-based chain elongation in neutral and slightly acidic environments and with a wide range of by-products, opening a window of possibilities for co-culturing.

### 3.3. Bioreactor study at pH 6.5 doubling initial lactate and acetate concentrations

In view of the fact that the production of MCFAs is similar at the three pH values studied, except for some kinetic differences, it was decided to carry out a fourth bioreactor experiment at the intermedium pH of 6.5. Furthermore, in all three of the above cases, bacterial activity stopped when all the initial lactate was consumed, so it was decided to change the fermentation strategy. In this way, it is also evaluated whether these substrate concentrations (i.e., 21.4 g/L) are high enough to exert an inhibitory effect on the microorganism. Therefore, keeping the same lactate:acetate molar ratio of 3:1 as in previous experiments, the initial substrate concentrations were doubled, thus starting from 17.2 g/L and 4.2 g/L of lactate and acetate, respectively (Figure 2D). Rapidly, the strain started to show some activity, with an increase of OD_600nm_ from 0.123 to 0.361 over the first 12 h of the experiment, while lactate and acetate concentrations decreased to 16.2 g/L and 4.0 g/L, respectively. The maximum OD_600nm_ of 2.1, was observed after 36 h, coinciding with the highest *n*-butyrate concentration reached, 1.1 g/L, while *n-*caproate and *n*-caprylate concentrations were 1.8 g/L and 0.25 g/L, respectively, at that point. The strain continued to produce MCFAs until 135 h after inoculation. Interestingly, as the initial substrate concentration was increased, it can be observed that *M. hexanoica* did not assimilate all the available lactate, unlike what appeared to happen when starting from 8.5 g/L. The use of higher initial substrate concentrations inevitably leads to higher toxicity exerted on *M. hexanoica*, which may restrict its performance. A solution to mitigate this issue could involve starting-up a continuous system, which inherently prevents the buildup of metabolites within the reactor. Indeed, at the end of the experiment, about 4.1 g/L lactate and 1.5 g/L acetate were still available, which corresponds to a consumption of 76 and 65%, respectively. With this approach, both *n*-caproate and *n*-caprylate selectivity could be slightly improved with respect to previous bioreactor studies (Table 2), and the maximum concentrations of *n*-butyrate, *iso*-valerate, *iso*-caproate, *n*-caproate, *n*-heptanoate and *n*-caprylate were, respectively, 1.1 g/L, 0.32 g/L, 0.28 g/L, 3.7 g/L, 0.15 g/L, and 1.5 g/L. To the best of our knowledge, this is the first time that *iso*-caproate production by *M. hexanoica* has been detected and quantified. Also, there was no evidence of *n*-caprylate formation from lactate and acetate alone by *M. hexanoica* reported so far in the literature.

**Table 2:** Summary of the production of MCFAs by *M. hexanoica* in all bioreactor studies conducted.

	Max. *n*C_6_ (g/L)	Max *n*C_8_ (g/L)	*n*C_6_ selectivity (% mol/L)	*n*C_8_ selectivity (% mol/L)	Conversion efficiency (% COD)
Reactor pH 6.5	1.92	0.69	54.5	13.4	84.7
Reactor pH 5.8	2.08	0.70	54.4	14.7	92.0
Reactor pH 7.2	2.06	0.62	55.3	11.8	87.4
Reactor double Lac^−^ + Ac^−^ (pH 6.5)	3.68	1.47	58.4	18.8	76.2

COD, chemical oxygen demand; Lac−, lactate; Ac−, acetate.

It is noteworthy that the concentrations of *n*-caproate achieved from lactate and acetate by *M. hexanoica* are not as high as those achieved by *C. kluyveri* from ethanol and acetate, reaching 11.8 g/L, with the latter (San-Valero et al., 2019). However, *C. kluyveri* is not characterized by producing large concentrations of *n*-caprylate, whereas *M. hexanoica* in this study produced the considerable amount of 1.5 g/L from lactate and acetate as the only carbon sources. The highest recorded *n*-caprylate concentration reported with *M. hexanoica* was 5.12 g/L with a selectivity of 81.62% (mol C/L), using a large number of substrates (i.e., lactate, fructose, acetate, *n*-butyrate and *n*-caproate) (Kang, 2022). This evidence highlights the remarkable capacity of this strain to produce longer chain carboxylates. Nevertheless, it is essential to acknowledge that the requirement for a significant quantity of *n*-caproate to generate *n*-caprylate entails additional steps and costs (e.g., its production from ethanol or lactate and other carboxylates). Therefore, the exploration of production from shorter-chain substrates (i.e., acetate) should remain under investigation, as demonstrated here, where its utilization has proven effective in achieving relatively high concentrations of *n*-caprylate. On the other hand, up to 1.2 g/L of *n*-caprylate was detected with this strain but using fructose as electron donor and a combination of acetate and *n*-caproate as electron acceptors (Jeon et al., 2016). Kang et al. (2022) also reported low concentrations of *n*-caprylate in the fermentation broth when using fructose and acetate and/or *n*-butyrate. In a study carried out by Wu et al. (2019) in a mixed culture batch bioreactor study, with a 3:1 lactate:acetate molar ratio and with the same initial substrate concentrations as in the first three experiments of this work, *n*-caprylate concentrations of 3.77 ± 0.61 mM (0.544 ± 0.088 g/L) were found, which are comparable to those obtained in this study. Besides, in a continuous bioreactor study using waste activated sludge, with a sludge retention time of 15 days, 1.48 g COD/L (0.606 g/L) of *n*-caprylate was detected (Wu et al., 2023).

On the other hand, relatively low but considerable concentrations of odd chains (i.e., *iso*-valerate and *n*-heptanoate) have been found in the four conditions explored in bioreactor. These concentrations may be the result of amino acid metabolism due to the use of such high concentrations of yeast extract (10 g/L), since *M. hexanoica* cannot use the acrylate pathway with the consequent formation of odd-chains.

### 3.4. H_2_ and CO_2_ production

**Figure 3:**
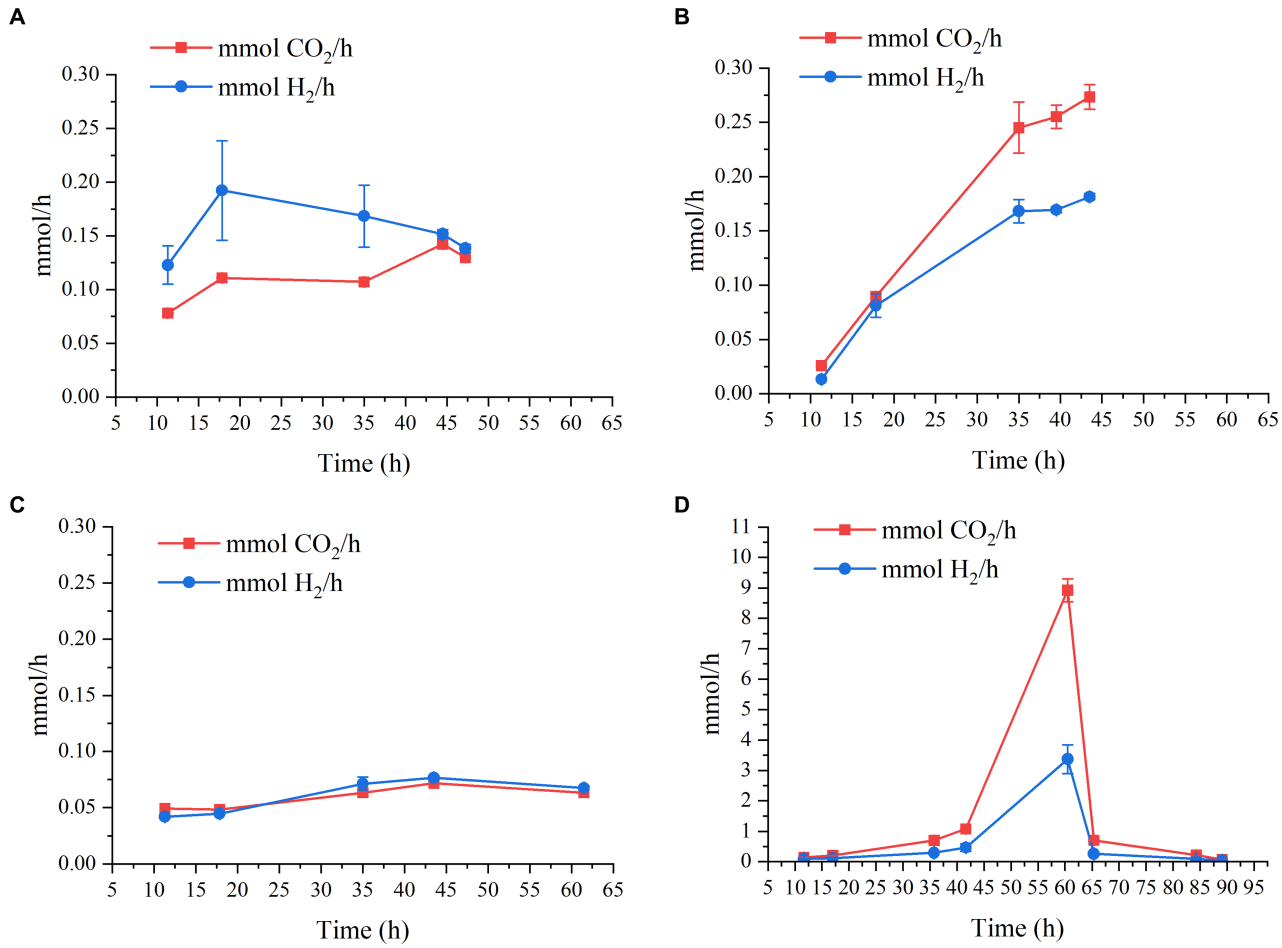
Production rates in mmol/h of CO_2_ and H_2_ as a function of time by *M. hexanoica* in the four bioreactor studies: **(A)** pH 6.5; **(B)** pH 5.8; **(C)** pH 7.2; **(D)** pH 6.5 with double substrate of lactate and acetate.

Regarding gas production, CO_2_ and H_2_ productivities as a function of time of the four bioreactor experiments can be seen in Figure 3. This graph shows that, regardless of pH, when the initial lactate and acetate concentrations are constant (i.e., 9 g/L and 2 g/L, respectively), H_2_ and CO_2_ production rate is quite similar, being slightly lower at pH 7.2, and below 0.28 mmol/h (Figures 3A–C). However, when lactate and acetate concentrations are doubled (i.e., 18 g/L and 4 g/L), both H_2_ and CO_2_ production soar to maximum values of 3.4 ± 0.47 mmol/h and 8.9 ± 0.37 mmol/h, respectively (Figure 3D).

Consequently, changing the fermentation strategy not only improved *n*-caproate and *n*-caprylate production especially as expected, but also increased CO_2_ and H_2_ production rate by almost 30-fold. During the chain elongation process from lactate and acetate, the more lactate that is available, the more acetyl-CoA will be formed, which in turn will result in a greater release of CO_2_. This is consistent with the conversion efficiency values calculated as indicated in Eq. 3 and shown in Table 2. As can be seen, in Eq. 3 the CO_2_ produced has not been considered, and consequently, the lowest conversion efficiency of 76.2% takes place when doubling the carbon input via lactate and acetate.


(3) $$\left(\frac{\sum {\left[\mathrm{carboxylates}\right]}_{\mathrm{produced}}}{{\left[\mathrm{lactate}\right]}_{\mathrm{consumed}}+{\left[\mathrm{acetate}\right]}_{\mathrm{consumed}}}\right)\frac{\left(g\frac{\mathrm{COD}}{L}\right)}{\left(g\frac{\mathrm{COD}}{L}\right)}\times 100$$

This has the implication that, although doubling the initial substrate concentration results in improved final concentrations of certain MCFAs, there is a considerable portion of carbon that is being diverted to CO_2_ release, rather than being harnessed for MCFA formation. This may be of particular interest and importance when it comes to experimental design. For example, to ensure minimal CO_2_ emissions during fermentation in a continuous bioreactor study, it would be advisable to feed a relatively low lactate concentration (below 18 g/L). Also, the CO_2_ and H_2_ produced by the strain can be used for other bioprocesses, such as fermentation by acetogenic bacteria (Fernández-Naveira et al., 2017; Arslan et al., 2022). In this scenario, it is likely that the amount of H_2_ produced would not be sufficient, so it would be needed to have some external supply. This is the first time that H_2_ and CO_2_ production by *M. hexanoica* has been studied, therefore, no data on the percentage of H_2_ and CO_2_ produced by *M. hexanoica* are available in the literature. However, H_2_ and CO_2_ concentrations in the headspace of reactors inoculated with a mixed culture and carrying out lactate-based chain elongation, have been found to remain around 20% and above 40% over time, respectively (Wu et al., 2018). On the other hand, H_2_ supplementation in a chain elongation reactor inoculated with sludge from lactate and acetate appeared to promote the reduction of propionate formed in the broth trough the acrylate pathway into propanol (Wu et al., 2019), which could be used again in the reverse *β*-oxidation pathway for the production of odd-MCFAs. Also, the authors postulate that CO_2_ released as a consequence of the transformation of pyruvate into acetyl-CoA during chain elongation from lactate could react with H_2_ (another by-product) to form acetate and then be reduced with H_2_ to yield ethanol, which could be used, respectively, as electron acceptors and electron donors for the formation of *n*-caproate and *n*-caprylate. Interestingly, the authors who isolated *M. elsdenii* found that the use of an electron donor slightly more oxidized than lactate, such as pyruvate, changes the metabolism of the strain from H_2_-producing to H_2_-consuming (Elsden and Lewis, 1953). *M. hexanoica* is known to use pyruvate (Jeon et al., 2017), however, no studies have been undertaken to ascertain whether it exhibits the same behavior as *M. elsdenii*, thereby here it is also proposed as a future field of study.

Overall, in this work, *M. hexanoica* demonstrates to be able to perform lactate chain elongation, thus producing MCFAs as well as H_2_ and CO_2_ with high activity in a pH range of at least 5.8–7.2. pH values outside this range were not explored given the pH range of 5.5–7.5 for theoretical growth specified by Jeon et al. (2017). Other chain elongating microorganisms, such as *C. kluyveri* have an optimum growth pH close to neutrality (i.e., 6.8) and show serious difficulties to grow if deviating much from this value. For example, this strain was shown to be able to produce *n*-butyrate and *n*-caproate from ethanol and acetate in co-culture with *C. aceticum* at pH 7.5, although it failed to maintain its activity in a prolonged manner, whereby OD_600 nm_ decreased abruptly and not even a pH reset to 6.8 was able to recover the activity of *C. kluyveri* (Fernández-Blanco et al., 2022). Similarly, an overly acidic pH of 5.7 proved to be detrimental to the chain elongating activity of *C. kluyveri*, producing low rates of MCFAs production in co-culture with *C. ljungdahlii* (Richter et al., 2016). On the other hand, *M. elsdenii* showed to be less sensitive to changes in pH than *C. kluyveri* since it maintained independent growth in lactate at pH (μ = 0.66 h^−1^) in the pH range of 5.0 to 6.6, whereas at a lower pH of 4.65 growth decreased substantially (μ = 0.17 h^−1^) (Weimer and Moen, 2013). Therefore, an advantage of *M. hexanoica* is that it can perform chain elongation with high activity in a range of at least 1.4 pH units, from 5.8 to 7.2, unlike other chain elongating strains. Furthermore, the major product of lactate fermentation by *M. hexanoica* was shown to be *n*-caproate, a reaction already reported to be thermodynamically favorable, either using acetate or *n*-butyrate as electron acceptor (Cavalcante et al., 2017; Kang et al., 2022).

As future research with this topic, it is proposed to try to improve the selectivity of the process towards a specific product. For example, it has been proven that higher electron donor:electron acceptor ratios favor the appearance of longer chains (Spirito et al., 2018). It is therefore suggested to explore different experimental conditions, such as varying the lactate:acetate molar ratio and also to encourage the use of this strain in co-culture with other strains of possible interest for the production of MCFAs, thanks to the range of pH values that it can withstand. Exploring the utilization of substrates like pyruvate and investigating whether the strain can effectively harness the produced H_2_ as a co-electron donor is another intriguing avenue for research. Still, it is worthy to note how *M. hexanoica* produces odd-chains even in small concentrations, from only lactate and acetate. Considering that the YE concentration used is quite high (i.e.,10 g/L), the assumption that the odd-chain fatty acids obtained after fermentation may come from amino acid metabolism can be made. In this context, it is conceivable that reducing the YE concentration could enhance selectivity for even-chain fatty acids by minimizing the presence of amino acids with odd-carbon chains. This reduction would potentially decrease the release of odd metabolic intermediates that favor the production of compounds like *n*-valerate and *n*-heptanoate.

## 4. Conclusion

This study highlights the potential of *M. hexanoica* as a chain elongator, using lactate and acetate, to produce MCFAs. The most cost-effective medium included YE, lactate and acetate as carbon sources. *M. hexanoica* performed well over a wide pH range (5.8, 6.5 and 7.2) with high conversion efficiencies, making it versatile for co-culture. This distinguishes *M. hexanoica* from other chain elongators, e.g., *C. kluyveri*, which is very susceptible to pH changes. From 17.2 g/L lactate and 4.2 g/L acetate, the major compound obtained was *n*-caproate (3.7 g/L), followed by *n*-caprylate (1.5 g/L), and *n*-butyrate (0.5 g/L), along with minor amounts of *iso*-valerate, *n*-valerate, *iso*-caproate, *n*-caproate and *n*-heptanoate. This is the first time that concentrations of *n*-caprylate and *iso*-caproate have been quantified from lactate and acetate with this strain. Thus, it is a microorganism that deserves further research and that allows upgrading lactate, which can be a by-product of different food and beverage industries, as well as cosmetics, towards MCFAs, such as *n*-caproate and *n*-caprylate, which are of great interest in the food, chemical, pharmaceutical and biofuel production industries. Also, the H_2_ and CO_2_ produced by *M. hexanoica* during these fermentations could be utilized in the formation of short-chain fatty acids and bioalcohols by other acetogenic strains. In this way, a better use of available resources could be achieved and waste or by-products from other industries could be converted into high value-added products.

In summary, this work aims to better understand the behavior of *M. hexanoica*, thus contributing to the state of the art.

## Data availability statement

The raw data supporting the conclusions of this article will be made available by the authors, without undue reservation.

## Author contributions

CF-B: Data curation, Investigation, Writing – original draft, Writing – review & editing. MV: Funding acquisition, Resources, Supervision, Writing – original draft, Writing – review & editing. CK: Data curation, Funding acquisition, Resources, Supervision, Writing – original draft, Writing – review & editing.
